# Large orb-webs adapted to maximise total biomass not rare, large prey

**DOI:** 10.1038/srep14121

**Published:** 2015-09-16

**Authors:** Aaron M. T. Harmer, Philip D. Clausen, Stephen Wroe, Joshua S. Madin

**Affiliations:** 1 Department of Biological Sciences, Macquarie University, North Ryde, NSW 2109, Australia; 2 Institute of Natural and Mathematical Sciences, Massey University, Albany, Auckland 0632, New Zealand; 3 School of Engineering, University of Newcastle, Callaghan, NSW 2308, Australia; 4 School of Environmental and Rural Sciences, University of New England, Armidale, NSW 2351, Australia

## Abstract

Spider orb-webs are the ultimate anti-ballistic devices, capable of dissipating the relatively massive kinetic energy of flying prey. Increased web size and prey stopping capacity have co-evolved in a number orb-web taxa, but the selective forces driving web size and performance increases are under debate. The rare, large prey hypothesis maintains that the energetic benefits of rare, very large prey are so much greater than the gains from smaller, more common prey that smaller prey are irrelevant for reproduction. Here, we integrate biophysical and ecological data and models to test a major prediction of the rare, large prey hypothesis, that selection should favour webs with increased stopping capacity and that large prey should comprise a significant proportion of prey stopped by a web. We find that larger webs indeed have a greater capacity to stop large prey. However, based on prey ecology, we also find that these large prey make up a tiny fraction of the total biomass (=energy) potentially captured. We conclude that large webs are adapted to stop more total biomass, and that the capacity to stop rare, but very large, prey is an incidental consequence of the longer radial silks that scale with web size.

Orb-web spiders evolved more than 200 mya^1^ and there is strong evidence for repeated evolution towards larger body sizes and high-performing silks and webs^e.g.^ 2,3,4,5. However, the selective forces driving evolution towards increasingly high prey-stopping capacity remain the subject of debate. Venner and Cassis^6^ proposed that while the capture of relatively small prey may increase a spider’s survival, it is only through catching rare, but very large prey items, that a spider can successfully mature and reproduce. Blackledge’s^7^ meta-analysis of 38 prey survey studies provides further support for the “rare, large prey” hypothesis. It suggests that although large prey make up only 17% of the total number of prey captured by spiders, they contribute 85% of the total biomass. This apparent importance of large prey to a spider’s energetic intake and subsequent fitness suggests an important role in shaping the evolution of high-performing webs^4^,^8^. That is, if total prey biomass is critical to spider fitness, and the bulk of that biomass is derived from rare, large prey, then the ability to stop these high kinetic energy prey should be the major force driving the evolution of web stopping performance.

Recently however, Eberhard^9^ questioned the validity of the “rare, large prey” hypothesis as a selective force driving the evolution of high-performing orb-webs. He suggested that the value of large prey has been greatly overestimated^6^,^7^ by applying Schoener’s^10^ equation to calculate insect mass from body length. Further, among large prey that are actually stopped by a web, there may be a bias towards insects with low body mass relative to size, or low flight velocities (e.g. long thin crane flies), as these will have lower kinetic energies and are more likely to be stopped. While there is a consensus that spider fitness is highly dependent on the biomass of captured prey, rather than absolute prey numbers^9^, the relative contribution of rare, large prey to a spider’s total captured biomass remains unclear.

Here, we combine ecological data with biophysical modelling of *Argiope radon* orb-webs, to investigate one of the major predictions of the rare, large prey hypothesis – that selection should favour webs with high prey-stopping capacity. We test (1) how web size relates to prey-stopping capacity; (2) how prey-stopping capacity of different sized webs translates into biomass potentially captured given the prey environment; and (3) the relative contribution of different sized prey to total stopped biomass. We aim to delineate the prey-stopping function of orb-webs from other critical and potentially competing web functions, such as interception of prey and prey retention. Each of these components of web function are likely to have conflicting selective effects that act in opposite directions on web characteristics and performance evolution^9^. Our finite element analyses indicate that larger webs have a greater prey-stopping capacity. However, ecological modelling also showed that very large prey make up a small fraction of a spider’s total stopped biomass, largely due to their relative scarcity. Large orb-webs appear to be adapted to maximise the total biomass stopped.

## Results and Discussion

We sampled insects in the field and found that both mass and kinetic energy of prey occurring sympatrically with the large orb-web spider *Argiope radon* were exponentially distributed, as found in other prey surveys^e.g.^ 7. The mean mass and flight velocity of haphazardly sweep-netted insects (*n* = 40) was 91.2 (±11.5 SE) mg and 1.3 (±0.1 SE) ms^−1^ respectively, with corresponding kinetic energy of 103.6 (±18.9 SE) *μ*J. Our measured prey velocities were lower than those recorded previously for the same insect orders^11^. As we aimed to investigate prey-stopping capacity at the upper limits of web performance and how this translates into biomass of potentially stopped prey, we applied an adjustment factor of 1.73 (calculated from Dean^11^) to estimate the upper values of prey kinetic energy likely experienced by *A. radon*. Both the measured and adjusted values were compared in models of total stopped prey biomass. The insect orders collected were Diptera, Odonata, Hymenoptera, Lepidoptera, Orthoptera and Hemiptera.

**Figure 1:**
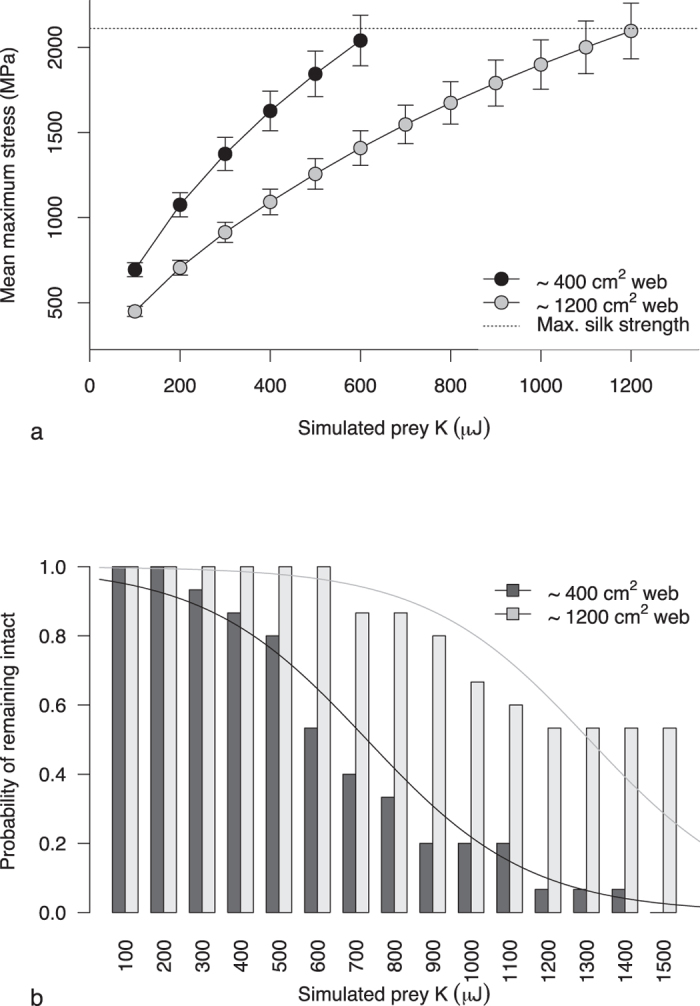
Silk stress and web breakage generated during finite element analyses. (**a**) Mean maximum stress (±SE) generated in FE models of an average small web and an average large web, across increasing simulated prey energies. Note that web performance was modelled up to a prey energy of 1500 μJ, however, data above the breaking strength of silk fibres are not shown as they are not biologically meaningful. (**b**) Probability of the web remaining intact after impact with increasing prey energies. Curves were fitted using logistic regression with logit function.

Finite element analyses (FEA) based on empirically derived silk and web properties (see Methods) showed that larger webs are able to absorb significantly more prey kinetic energy before breaking than smaller webs (Fig. 1), despite no detectable differences in silk mechanical properties (Table 1). Small webs (~400 cm^2^) reached their maximum stress at a FEA-simulated prey kinetic energy of 600 *μ*J, while large webs (~1200 cm^2^) did not reach maximum stress until 1200 *μ*J. Predictably, the probability of a web remaining intact after impact decreased significantly with increasing prey kinetic energy (z = −10.2, *p* < 0.001; Fig. 1B). Also, large webs were significantly less likely to break at a given prey energy (z = 8.2, *p* < 0.001). Our results show that web architecture plays a significant role in determining web performance when prey kinetic energy is high and pushes webs to their performance maxima. As radials absorb the majority of prey energy^12^, the longer radial threads in larger webs account for increases in the amount of kinetic energy that can be absorbed compared to smaller webs. The importance of radial length in energy dissipation is further supported by the fact that larger webs were more sparsely meshed (Table 1). While the denser mesh in smaller webs is a potential consequence of spiders fitting their webs to a smaller space^13^,^14^, denser meshes did not seemingly increase the energy-absorbing capacity of smaller webs through either additional material or aerodynamic damping (Fig. 1, Table 1). Mean maximum stress at breaking in small webs was half that of larger webs, which matches with the approximate halving of radial length in smaller webs, suggesting radial length is the dominant factor determining maximum energy-absorbing capacity. Sensenig *et al.*^4^ found a compensatory relationship between silk quality and quantity across orb-web spider species, suggesting that silk material properties are fine-tuned to web architectures. However, we found that silk properties do not vary with web size in *A. radon*, and therefore demonstrate that silk quantity/quality trade-offs are not apparent within this species. Furthermore, we found that the stress generated during a prey impact was highly localised to the radial of impact across web sizes, with a low degree of recruitment of neighbouring threads (consistent with Cranford *et al.*^15^, although see Sensenig *et al.*^12^). Peak stress was significantly higher in the radial of impact compared to the two nearest radials in both smaller and larger webs (small webs, t_14_ = 13.99, *p* < 0.001; large webs, t_14_ = 7.78, *p* < 0.001). It is therefore unlikely that the sparser mesh found in larger webs significantly influenced prey stopping ability.

**Figure 2:**
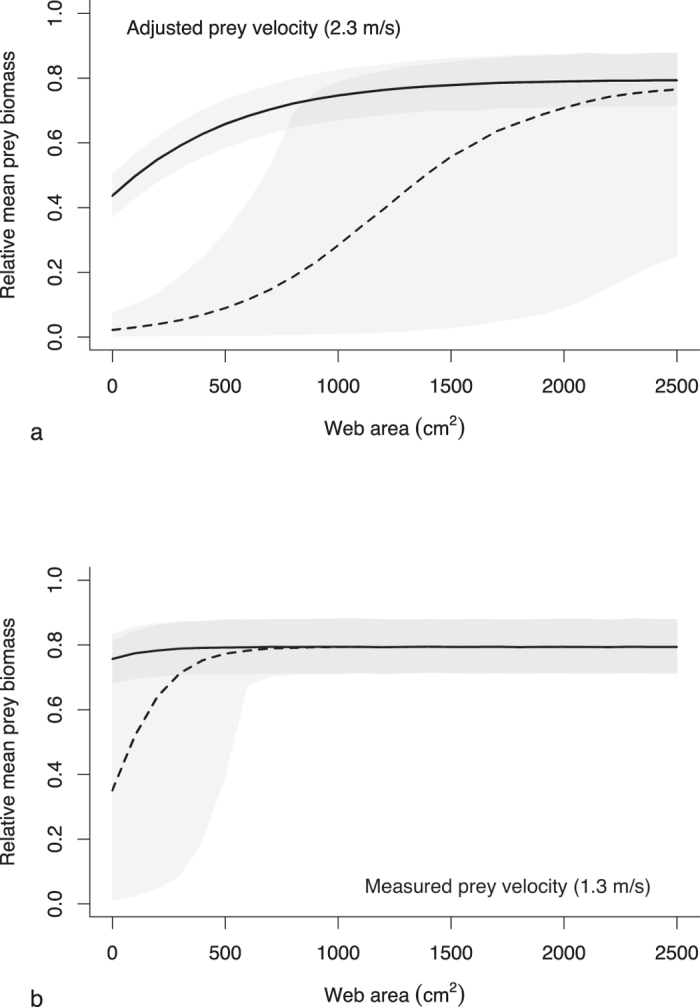
Mean relative biomass stopped across increasing web sizes. Biomass was calculated by summing the mass of prey items predicted to be stopped by a web. The solid lines indicate the “best case” scenario, where a web continues to function after a prey item has broken through the web (i.e. sum of all prey predicted to not break the web). The dashed lines are the “worst case” scenario, where a web is assumed to be completely non-functional once a prey item has broken the web (i.e. sum of all prey up to the first prey predicted to break the web). Shaded areas are 95% prediction intervals. (**a**) Mean total stopped biomass using adjusted prey velocities. (**b**) Mean total stopped biomass using measured prey velocities.

We modelled the prey biomass stopped per unit area of web across web sizes by using our FEA results to predict the success of stopping prey randomly sampled from the distribution of empirically derived prey kinetic energies. Our prey capture modelling indicates that stopped biomass per unit area of web increases with total web area. We first consider biomass stopped at the upper extreme of prey kinetic energies likely experienced by spiders (using our *adjusted* prey velocities) (Fig. 2A). In the “best case” scenario—where webs remain functional after encountering prey that would break the web—mean biomass stopped plateaus at web areas of approximately 1200 cm^2^. Of note, the mean web area for adult female *A. radon* measured in the field is 1150 cm^2^ 16. In the “worst case” scenario—where webs lose all functionality after encountering prey that would break the web—mean biomass stopped plateaus at approximately 2500 cm^2^, the approximate maximum web area measured in the field^16^. Variation in prey stopped is greater in the “worst case” scenario because there can be cases where a web encounters a large prey and loses functionality soon after it is built. Greater biomass stopped per unit area in larger webs follows from the fact that larger webs can absorb greater kinetic energy, and so are more likely to stop larger prey. The advantage of larger webs may be two-fold if larger webs do indeed intercept more prey, and retain that prey after stopping its momentum. However, as silk was more sparsely distributed in larger webs, prey retention times may be reduced compared to smaller webs with more closely packed radial and capture spiral threads, as finer meshed webs retain prey longer^17^. While more sparsely meshed webs may be compensated for with stickier viscid glue droplets, larger webs with sparser meshes may also intercept fewer small prey if they are able to fly through the web without contacting any sticky spirals.

When mean prey kinetic energies are lower (using our *measured* prey velocities), mean total biomass stopped per unit area of web does not vary as strongly with web size (Fig. 2B). In the “best case” scenario, biomass stopped per unit area is consistent across web sizes. In the “worst case” scenario, biomass stopped only drops off in very small webs (<500 cm^2^). While stopped biomass does not vary strongly with web size when prey kinetic energies are generally low, spiders are still faced with the trade-off between building larger webs to maximise interception rates, and building densely meshed webs to maximise retention times. The consistency in stopped biomass across web sizes when mean prey energies are low (relative to the performance maxima of webs) suggests that architecture is less important in very high-performing webs such as those built by *A. radon*. In smaller species with lower-performing silks, webs will be pushed closer to their performance maxima and so web size will likely play a much greater role in determining stopped biomass, as seen when we increased prey velocities in our model.

**Figure 3:**
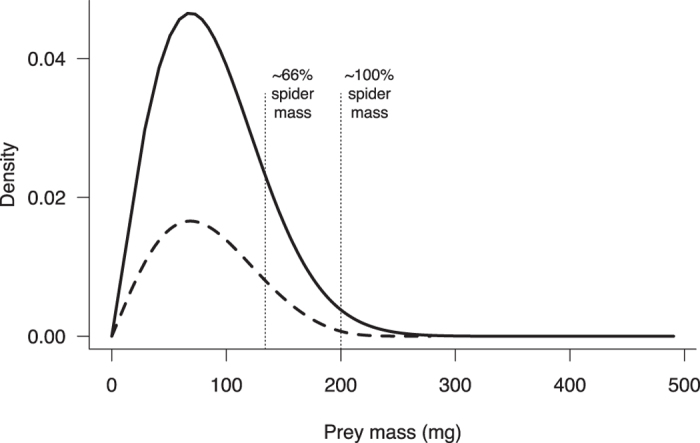
Distribution of total prey biomass stopped by a spider for average sized webs in prey capture simulations. Bold line shows values for a mean sized web using measured prey velocities, dashed line shows values for a mean sized web using adjusted prey velocities. Vertical dotted lines show the proportion of prey greater than 66% and 100% of mean spider body mass.

Eberhard^9^ argues that previous studies have greatly over estimated the value of rare, large prey by applying Schoener’s^10^ equation to estimate prey mass, and therefore contends that total prey biomass, and not necessarily rare, large prey, is the most important factor driving web evolution. A potential problem in assessing the importance of prey size distributions is defining what constitutes “large” prey. To date, such decisions have been essentially arbitrary, for example, large prey have been defined as greater than 66% of spider body length^7^, and greater than approximately 100% of spider body length^6^. Here, the distribution of stopped prey mass across the range of adult female webs measured in the field show that the majority of biomass is derived from prey with mass less than 200 mg (Fig. 3). Using for example the value of 66% (mass instead of body length), we find that so-called large prey comprise approximately 13–15% (adjusted vs. measured) of stopped biomass in an average sized *Argiope radon* web. Using the more conservative value of prey mass greater than 100% of spider mass, the contribution of large prey to total biomass drops to <1–1.7% (adjusted vs. measured). While the size distributions of prey stopped by webs during simulations differ significantly when using measured versus adjusted prey velocities (Two-sample Kolmogorov-Smirnov test; D = 0.43, *p* < 0.001), both distributions are strongly right-skewed (Fig. 3). These results indicate that rare, large prey contribute far less to a spider’s total stopped biomass than previous studies have suggested (e.g. 85%^7^).

Our results are robust compared with previous studies. We directly measured prey mass and flight velocity, rather than calculating mass from body length, and subsequently support Eberhard’s assertion that Schoener’s equation greatly overestimates prey mass. The reason we find large prey contribute so little to total biomass is that they are encountered too infrequently. While it is possible that we underestimated the frequency of rare, large prey in the environment or overestimate the capture of small prey, only a fundamental change to the shape of the prey biomass distribution would support the “rare, large prey” hypothesis (e.g., from exponential to normal or uniform distribution). Finally, while direct sampling from a spider’s environment may not perfectly represent the prey actually “available” to spiders^18^ —i.e. if there is a bias in prey size or type caught by webs—the fact so few “large” prey were sampled at all means that it is unlikely we underestimated their relative importance.

Larger, higher-performance webs stop more total biomass attributable to a reduction in the skew of the prey size distribution, rather than “rare, large” prey specifically. Other facets of prey capture may benefit from increased prey-stopping capacity, such as less damage incurred during high-energy impacts, but our results demonstrate that maximising stopped biomass may be the main factor driving the evolution of high-performing webs. Despite our single species focus, we have demonstrated a potential selective pathway for the evolution of high-performing webs—increasingly large webs with longer radial threads have increased stopping capacity and subsequently increased stopped biomass. As total biomass is critical to fecundity^6^,^9^, larger webs may provide spiders with a selective advantage.

## Methods

### Spiders, web architecture and silk properties

We studied silk and web properties in the large tropical orb-web spider, *Argiope radon* (Araneidae). These spiders are common in the Katherine region in the Northern Territory, Australia, and are typically located along river banks. Webs are usually attached to vegetation overhanging the water, or within 1–2 m of the water’s edge. We collected adult female *A. radon* from the banks of the Katherine River at Manbulloo Homestead (−14.517382°, 132.197514), approximately 10 kms SW of Katherine town centre. Typical riparian vegetation consists of grasses, low shrubs and trees, including genera such as *Paspalum, Pandanus*, *Melaleuca*, *Eucalyptus* and *Barringtonia*. The climate is semi-arid tropical savanna with distinct wet and dry seasons. Mean daily high and low temperatures in August when spiders were collected are 32 °C and 15 °C. Mean monthly rainfall in August is 1.3 mm (266 mm in January). Spiders were returned to Macquarie University and housed in a temperature control room (~25 °C) on 12 hour light/dark cycle. They were fed 2–3 times weekly with large house crickets (*Achetus domesticus*) and watered daily with a mist sprayer.

**Figure 4:**
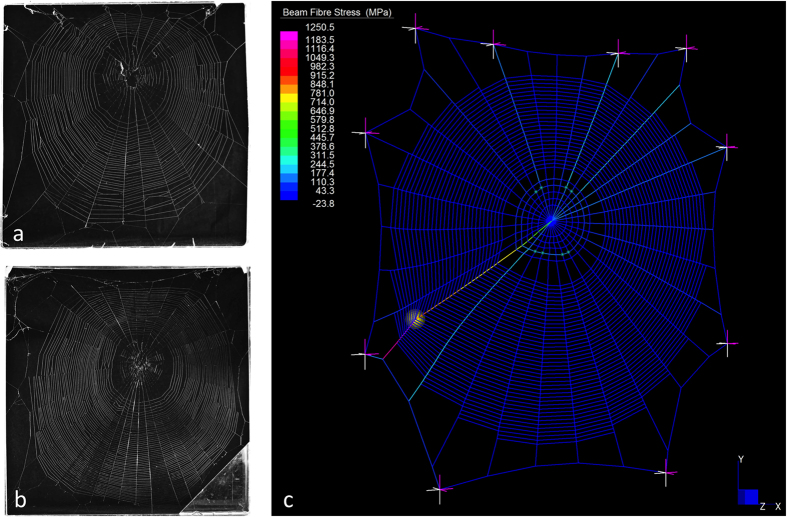
Example webs built in small and large frames and example finite element model. (**a**) Web built in a 25 × 25 cm frame. (**b**) Web built in a 50 × 50 cm frame by the same spider. (**c**) Finite element model of an average web built in a 50 × 50 cm frame.

To measure web architecture and silk tensile properties for different web sizes, spiders (N = 16) were provided with either a small (25 cm × 25 cm) or large (50 cm × 50 cm) acrylic frame for web-building (Fig. 4A,B). Webs were photographed against a black background and we collected radial and capture spiral silk samples for tensile testing (see below). Spiders were then provided with the alternate sized frame in which to build their webs, and web and silk properties were recorded again. The order of frame treatment (small or large) was randomised.

We measured web characteristics from the photographs, including vertical and horizontal diameters of the capture area and hub, radial number, capture spiral turns and spacing above and below the hub, using ImageJ (vers. 1.46r). From each web we collected four sections of capture spiral and four radials, one from each of the cardinal points. Silk was collected on C-shaped pieces of cardboard across a 15 mm span and secured with cyanoacrylate (as per Sensenig *et al.*^4^). Each silk sample was photographed (Canon EOS 600D on a Motic BA300 polarising light microscope) at three points along its length, its diameter measured using ImageJ, and the cross sectional area of the double stranded thread was calculated.

Silk tensile properties were measured using an Instron 5542 materials testing machine with a 0.5 N load cell (sensitive to ±2% of measured values). Each sample was stretched at a rate of 1 mm s^−1^ until breaking, with force and extension recorded every 100 ms. Force-extension curves and fibre cross-sectional areas were used to calculate stress, strain, Young’s modulus and toughness (area under stress strain curve). Fibres were assumed to have constant volume during stretching^19^.

### Prey kinetic energy in the field

We filmed insects flying inside a flight tunnel in the field to calculate the flight velocities and kinetic energies of prey sympatric with *A. radon*. The flight tunnel consisted of a 1 m length of U-shaped plastic drainage pipe (15 cm × 15 cm) with a clear plastic top and a 100 ml collecting vial at each end. A 1 cm black and white grid was painted on the base to provide a scale. A high-speed video camera (Casio Exilim EX-F1; 300 frames per second) was set up above the tunnel and recorded insect flights. To encourage insects to fly on a direct path through the tunnel, the entire setup was covered in black cloth so that only a small section at one end was exposed to bright light. Insects (N = 40) were caught by haphazard sweep netting around the river banks in the same locations that spiders were collected. To sample prey that might fly into a web, insects were sweep-netted only when they flew within reach of a fixed position, rather than actively pursuing them along the river banks. Multiple fixed sweep-netting positions were used. When an insect was caught it was released into the dark end of the tunnel and its flight recorded as it flew towards the bright end. The mass (*m*) of each insect was recorded using a balance and its flight velocity (*v*) measured using ImageJ. These values were then used to calculate prey kinetic energy as 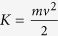.

We compared our measured insect flight velocities with similar measurements for maximum flight speeds from the literature^11^. Our observations appeared to consistently underestimate maximum insect flight velocity (possibly due to the short length of the flight tunnel), and we therefore applied an adjustment factor of 1.73 to our measured values. The correction factor was calculated by comparing the mean flight velocity of each of our observed insect orders with the mean flight velocity for the same insect orders presented in a comprehensive review of insect flight velocities^11^. We only compared values for the same insect orders that we recorded and for studies that used a comparable flight tunnel/chamber technique.

### Finite element analysis of web performance

We constructed finite element models of an average small web and average large web to investigate the prey stopping capability of different web sizes. Mean web dimensions, radial numbers and spiral spacing were based on values measured in photographs of webs built in small and large frames in the laboratory. Mean silk properties (Table 1) corresponding to these same webs were also applied to FE models. Models were constructed using Strand7 v2.5 finite element software. Silk fibres were treated as cut-off bars and a static spider mass (200 mg; divided over 8 contact points) was added at the hub. Silk pretensions (estimated from Wirth and Barth^20^) and gravity preconditions were applied to the models. Pretension values for frame, radial and capture spiral threads were 2.32 mN, 0.33 mN and 0.01 mN, respectively. While we have used average web dimensions to create ‘standardised’ web models (e.g. Fig. 4C), performance did not vary noticeably from web models generated by digitally tracing the structure to create an exact replica (AMTH, unpublished data). The lack of difference between ‘standardised’ and more realistic webs is likely due to the fact that stopping performance is driven by radial length, with negligible contribution from the capture spiral^12^.

Prey impacts were simulated in non-linear dynamic analyses using a non-structural mass (i.e. independent from the web structure) to simulate the prey, and a zero-gap uptake beam (allows the mass to be ‘launched’ at the web structure at a specific point) to impact the prey onto the web at 90° to the web plane. An initial velocity of 2 m s^−1^ was applied to the mass. Prey impacts were simulated across masses from 50–750 mg (in 100 mg increments), corresponding to kinetic energies of 100–1500 μJ, approximating the range calculated from prey in the field. For each web size and prey energy, we simulated a prey impact at 15 different randomly selected nodes within the capture area to account for variation in prey-stopping ability in different parts of the web. We recorded the maximum stress generated in the web and whether or not stress exceeded the maximum breaking stress of web fibres during simulated impacts. The probability of breaking with increasing input energy was compared between small and large webs using logistic regression with logit link function (R^21^; ‘stats’ package).

### Modelling stopped biomass

To understand how prey size distribution and web performance translate to total biomass stopped, we simulated prey encounters across web sizes using randomised sampling of prey from the distributions of measured and adjusted prey energies. Simulations were performed in R^21^. For web sizes spanning the range of naturally occurring webs, 100 “prey” were randomly sampled, with replacement, from the distribution of prey kinetic energies measured in the field. The total biomass of caught prey was then summed based on the probability of a given prey item being stopped by a web of a given size. The probability of prey being stopped by a given web was determined from the generalised linear model comparing stopping ability in the FE models of average webs. To estimate 95% prediction intervals, randomised sampling to calculate total biomass in each web size was run 10,000 times. We did not include running cost to reach prey in our model as this is negligible relative to the investment cost of web construction. Peakall and Witt^22^ show that for *Araneus diadematus*, cost of running to reach prey is only ~1.6% of the cost of building a web. Total stopped biomass was modelled in two scenarios; 1) best case: webs were assumed to be still functional if a prey item broke through the web; and 2) worst case: webs were assumed to be completely non-functional once a prey broke through the web. In other words, in case 1, all prey below 50% probability of breaking the web were assumed to be caught and their biomass summed, and in case 2, prey masses were only summed until the first prey item was encountered that exceeded 50% probability of breaking the web. In reality, the degree of web functionality after damage would lie somewhere between these two scenarios. Recent modelling indicates that an orb-web remains largely functional after damage due to the non-linear nature of silk tensile properties^15^, as well as the fact that spiders rapidly repair damage^23^.

## Additional Information

**How to cite this article**: Harmer, A. M. T. *et al.* Large orb-webs adapted to maximise total biomass not rare, large prey. *Sci. Rep.* **5**, 14121; doi: 10.1038/srep14121 (2015).

## Figures and Tables

**Table 1:** Mean (±SE) web architecture properties and silk tensile properties for webs built in small and large frames.

	**Small frame**	**Large frame**	**Test statistic**	***P***
N	16	16		
Web area (cm^2^)	406.4 (21.0)	1143.9 (70.1)	V = 0	<0.001
No. radials	26.1 (0.8)	30.5 (0.9)	t = −4.72	<0.001
Radial length (cm)	10.7 (0.2)	18.3 (0.5)	V = 0	<0.001
Total radial length (cm)	292.1 (13.9)	607.3 (29.1)	t = −11.30	<0.001
Total capture spiral length (cm)	989.9 (72.3)	2468.8 (174.8)	t = −9.73	<0.001
Spiral spacing (cm)	0.3 (0.02)	0.4 (0.02)	t = −4.02	0.001
Relative silk/area (cm/cm^2^)	3.2 (0.1)	2.7 (0.1)	t = 3.64	0.002
Max. radial stress (MPa)	2043.7 (113.2)	2182.149 (124.6)	t = −0.89	0.39
Max. capture spiral stress (MPa)	181.9 (15.3)	189.7 (12.0)	t = −0.85	0.41
Radial strain (ln(*L*_1_/*L*_0_))	0.25 (0.005)	0.26 (0.008)	t = −1.08	0.30
Capture spiral strain (ln(*L*_1_/*L*_0_))	0.95 (0.03)	0.91 (0.03)	t = 1.41	0.18
Radial Young’s modulus (MPa)	8022.3 (903.5)	8074.3 (1182.2)	t = 0.44	0.67
Capture spiral Young’s modulus (MPa)	25.1 (3.9)	25.3 (3.7)	t = −0.60	0.55
Radial toughness (MJ/m^3^)	229.2 (26.0)	222.3 (15.4)	t = −0.02	0.98
Capture spiral toughness (MJ/m^3^)	24.7 (2.7)	25.2 (2.4)	t = −0.35	0.73

Means were compared using paired t tests (log_10_ transformed where data not normally distributed) or Wilcoxon signed rank tests (where data were not normal after transformation).
